# Ventricular lavage for post-haemorrhagic hydrocephalus

**DOI:** 10.1186/1743-8454-7-S1-S49

**Published:** 2010-12-15

**Authors:** Ian Pople, Andrew Whitelaw

**Affiliations:** 1 Department of Neurosurgery, Frenchay Hospital, Bristol, UK. Department of Neonatal Medicine, Southmead Hospital, Bristol, UK

## Background

Early results of an international RCT comparing drainage, irrigation &fibrinolytic therapy (DRIFT)for prematurity-associated post-ventricular dilatation (PHVD), with standard treatment suggested no benefit in terms of avoidance of shunt dependency. There was also an increased rate of secondary bleeds in those having DRIFT. The longer term benefits or otherwise of DRIFT treatment were unknown.

## Materials and methods

We randomly allocated 77 preterm infants with PHVD to either DRIFT or standard treatment (ie tapping off cerebrospinal fluid to control excessive ventricular expansion). Severe disability was assessed at 2 years’ corrected age.

## Results

Of 39 infants assigned to DRIFT, 21 (54%) died or were severely disabled versus 27 of 38 (71%) in the standard group (adjusted odds ratio 0.25 [95% confidence interval: 0.08–0.82]). Among the survivors, 11 of 35 (31%) in the DRIFT group had severe cognitive disability versus 19 of 32 (59%) in the standard group (adjusted odds ratio: 0.17 [95% confidence interval: 0.05– 0.57]). Median Mental Development Index was 68 with DRIFT and_50 with standard care.

## Conclusions

Despite inducing an increased rate of secondary intraventricular bleeding, DRIFT reduced severe cognitive disability in survivors and overall rates of death or severe disability. A modification of DRIFT involving simple ventricular lavage and fibrinolytic therapy (limited to unblocking clogged-up drainage catheters) will now be employed in clinical practice at our unit.

